# Pathomechanisms of Blood-Brain Barrier Disruption in ALS

**DOI:** 10.1155/2019/2537698

**Published:** 2019-07-10

**Authors:** Nicholas Kakaroubas, Samuel Brennan, Matthew Keon, Nitin K. Saksena

**Affiliations:** ^1^Neurodegenerative Disease Section, Iggy Get Out, 19A Boundary Street, Darlinghurst NSW 2010, Sydney, Australia; ^2^School of Biotechnology and Biomolecular Sciences, University of New South Wales (University of NSW), Chancellery Walk, Kensington NSW 2033, Sydney, Australia

## Abstract

The blood-brain barrier (BBB) and the blood-spinal cord barrier (BSCB) are responsible for controlling the microenvironment within neural tissues in humans. These barriers are fundamental to all neurological processes as they provide the extreme nutritional demands of neural tissue, remove wastes, and maintain immune privileged status. Being a semipermeable membrane, both the BBB and BSCB allow the diffusion of certain molecules, whilst restricting others. In amyotrophic lateral sclerosis (ALS) and other neurodegenerative diseases, these barriers become hyperpermeable, allowing a wider variety of molecules to pass through leading to more severe and more rapidly progressing disease. The intention of this review is to discuss evidence that BBB hyperpermeability is potentially a disease driving feature in ALS and other neurodegenerative diseases. The various biochemical, physiological, and genomic factors that can influence BBB permeability in ALS and other neurodegenerative diseases are also discussed, in addition to novel therapeutic strategies centred upon the BBB.

## 1. Introduction

The blood-brain barrier (BBB) is a highly selective, semipermeable complex that surrounds most of the blood vessels in the brain [1], except for the circumventricular organs (CVOs) centred around the ventricles of the brain. CVOs are characterized by their highly permeable microvasculature and are involved with sensory and secretory systems within the brain [2, 3]. The BBB separates the blood from the extracellular cerebrospinal fluid and protects the brain from bloodborne pathogens and toxins while allowing the diffusion of oxygen, carbon dioxide, and small lipophilic molecules/ethanol [4]. Maintenance of the BBB is essential for a tight control of the chemical composition of the brain's interstitial fluid (ISF) essential for synaptic function as well as offering a form of protection against bloodborne pathogens [1, 4]. Further separation of the central nervous system (CNS) from the cardiovascular system occurs via the blood-spinal cord barrier (BSCB). The BSCB acts as a functional equivalent of the BBB by maintaining the microenvironment for the cellular constituents of the spinal cord [5]. There is reason to believe that the permeability of the BBB and BSCB differs with evidence suggesting that the BSCB is more permeable [5]. This difference in permeability has attracted motor neuron disease (MND) research, with findings suggesting that the BSCB is damaged in human and rodent ALS sufferers. Despite this evidence, the BSCB (like the BBB) breakdown in disease pathogenesis remains unclear [6, 7].

There are three cellular components that make up the BBB: this includes the capillary basement membrane (BM), astrocyte end-feet ensheathing the vessels, and pericytes (PCs) embedded within the BM. Tight junctions (TJs) are present between the cerebral endothelial cells and serve the function of limiting the paracellular flux of molecules across the BBB. TJs appear as apparent sites of fusion involving the leaflets of plasma membrane of adjacent endothelial cells. Similarly, adherence junctions (AJs) are more basal than TJs and are defined by their cytoplasmic face being linked to the actin cytoskeleton [8]. It is currently accepted in scientific literature that all the components of the BBB are essential for normal function and stability of the BBB. Despite sharing the same principal building blocks with the BBB, there is evidence suggesting morphological and functional differences in the BSCB. The differences between the two barriers include an increased permeability to tracers: [3H]-D-mannitol and [14C]-carboxyl-inulin [9], and cytokines: interferon-*α*, interferon-*γ*, tumor necrosis factor-*α* [10], decreased Occludin and ZO-1 protein expression in TJ [11], and decreased VE-cadherin and *β*-catenin protein expression in AJ [11].

Neuroimaging studies have demonstrated early BBB dysfunction in Alzheimer disease (AD) [12], Parkinson disease (PD) [13], Huntington disease (HD) [14], amyotrophic lateral sclerosis (ALS) [6, 15, 16], and multiple sclerosis (MS) [17]. These demonstrations of BBB dysfunction have been confirmed with biofluid biomarker data and observations of postmortem tissue analysis [6, 12–17]. For the sake of this review, “hyperpermeability” will refer to an increased state of permeability in the otherwise semipermeable membranes. BBB hyperpermeability represents a significant area of research as, despite being continuously examined in multiple motor neuron diseases (MNDs) [18], the exact biochemical pathway of BBB breakdown is not yet known.

This review aims to examine current research understandings of BBB hyperpermeability and mechanisms of breakdown to identify key research targets and areas that may shed light on the mystery surrounding the pathophysiology of BBB (and BSCB) breakdown. The key points of this review revolve around the mechanisms involved in oxidative stress, cerebrospinal microbleeds, underlying genomic modalities, and the epidemiology of ALS, thereby providing insights into potentially valuable knowledge gaps. To conclude, this review will make recommendations to investigative areas that hold promise in understanding the mechanisms involved in BBB breakdown at the biological and molecular level and provide insights into therapeutic strategies to modulate BBB permeability.

## 2. Oxidative Stress

Radicals are defined as a molecule species with one or more unpaired electrons and are characterised by being highly reactive. Reactive Oxygen Species (ROS) are a group of oxygen centred radicals. ROS can damage the body through hypoxia and reoxygenation of cells. Hypoxia and reoxygenation of BBB endothelial cells cause an increase in the paracellular permeability [19, 20] of the structure, leading to a state of hyperpermeability. ROS may also cause changes in the localization and structure of Occludin [21], which may influence the regulation and assembly of tight junctions which are essential structures in the BBB [22].

ROS can be produced endogenously and taken into the body exogenously. Exogenous sources of ROS can be obtained from pollutants, tobacco smoke, xenobiotics, radiation (ultraviolet and ionizing), ozone, chemotherapeutic agents, pesticides (e.g., Aldrin/dendrin [23, 24]), organic solvents, alcohol, and some metals [25, 26]. Endogenous ROS are produced intracellularly and extracellularly with the major producers being NADPH oxidase (NOX), nitrogen oxide synthase, Xanthine oxidase, Heme proteins, Cytochrome P_450_, phagocytes, mitochondria, peroxisomes, and the endoplasmic reticulum [27–29].

The significance of ROS in the degradation of neuromuscular junctions in cases of MND, especially sporadic ALS (sALS), has been examined extensively within literature as a proposed mechanism of neuromuscular degeneration as per the “Dying Back hypothesis.” The Dying Back hypothesis however does not account for BBB or BSCB degradation. ROS has been demonstrated to cause BBB dysfunction through alcohol induced activation of myosin light chain (MLC) kinase and phosphorylation of MLC and TJ proteins [30]. Furthermore, BBB disruption has been shown to be mediated by oxidative stress through stimulation of inositol 1,4,5‐triphosphate (IP_3_R)‐gated intracellular Ca^2+^ release resulting in a similar MLC kinase activation. As such, oxidative stress remains a topic of interest in investigating BBB hyperpermeability [31].

The effects of an abundance of ROS can be mitigated by the metabolism of these free radicals through neutralisation via antioxidants [32]. Oxidative stress is when there is more ROS than antioxidants and can have many physiological effects on the body. There are enzymatic and nonenzymatic antioxidant systems in humans which appear to be key aspects in the fight against ROS [33].

### 2.1. Enzymatic Antioxidants

#### 2.1.1. Nrf2 Transcription Factor

It is worth noting the importance of the transcription factor, nuclear factor erythroid 2–related factor 2 (Nrf2), and its role in oxidative stress. The transcription factor is constitutively expressed in all tissues [34]. Nrf2 functions at the interface of cellular redox and intermediary metabolism reactions as the target genes are involved in antioxidant enzyme production [35–37]. Nrf2 is a basic region leucine zipper transcription factor [38] that forms heterodimers in the nucleus of cells which recognises the enhancer sequence known as antioxidant response element (ARE). ARE enhancer sequences are present in the regulatory regions of over 250 genes [39]. Not only involved in antioxidant production, decreased Nrf2 activity decreases the expression of key tight junction proteins such as occludin and claudin-18 [39].

Nrf2 is activated by sulforaphane [40]: an organosulfur compound found in cruciferous vegetables, e.g., broccoli and Brussels sprouts [41]. Animal studies have shown that sulforaphane administration after brain injury increased neuroprotective effects of Nrf2 [42]. For its role in ARE and tight junction proteins, Nrf2 represents a significant direction for ALS- or neurodegenerative disease-induced BBB disruption and hyperpermeability.

Supporting this, Kraft et al. (2007) evaluated the endogenous activation of the Nrf2-ARE system during the induction of pathology in mutant SOD mouse models and showed that Nrf2-ARE activation appears to progress in a retrograde fashion along the motor pathway, thereby implying the contributions of the Nrf2-ARE pathway and its activation which can persist throughout ALS pathology and which increases in concert with disease severity [43].

#### 2.1.2. SOD Protein Family

The superoxide dismutase (SOD) family of enzymes function to catalyze the dismutation of superoxide anions (O_2_^−^) [44, 45]. Mammals have three isoforms of SOD that require metal cofactors (Cu, Zn, or Mn) for proper activation. Superoxide anions are a reactive oxygen species and are a byproduct of the one-electron reduction of oxygen (O_2_) that occurs as an antimicrobial immune response employed by phagosomes. In the production of superoxide anions, the membrane associated, flavoprotein enzyme, nicotinamide adenine dinucleotide phosphate (NADPH) oxidase catalyses oxygen to produce superoxide anions and hydrogen cations [45]:

(1) $$\begin{array}{c} NADPH+2O_{2}↔{NADP}^{+}+2{O_{2}}^{-}+H^{+} \end{array}$$

**Figure 1:**
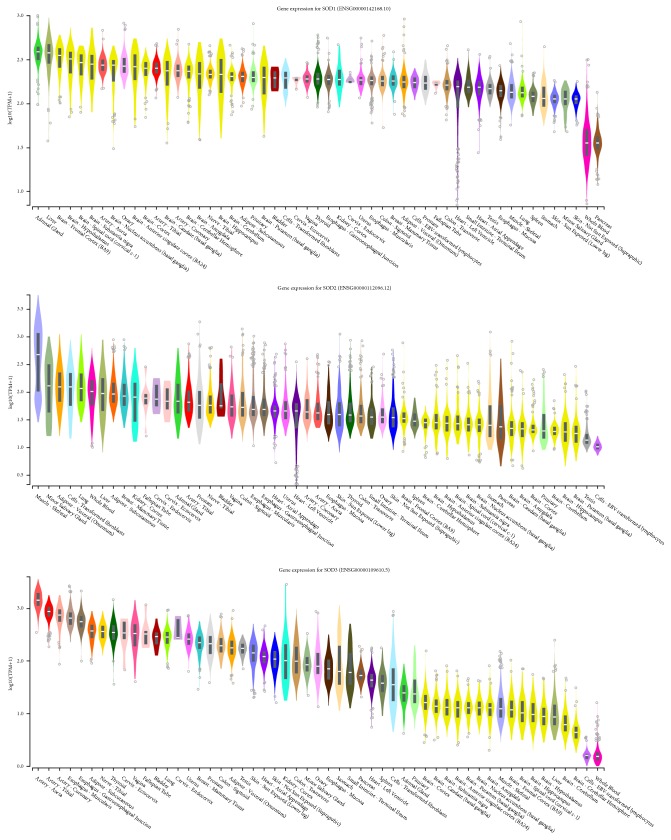
GTEx Analysis Release V7 Gene expression showing gene expressions of the SOD family of enzymes in TPM (Transcripts Per Million) represented logarithmically. SOD1 has high expression in regions of the brain, SOD 2 is uniform amongst somatic cells, and SOD3 has high expression in Aorta, Tibial, and Coronary arteries.

The SOD family of proteins act as one of many defence mechanisms against superoxide anions. The SOD protein family CuZn-SOD, Mn-SOD, and EC-SOD is encoded by the SOD gene family which includes SOD1, SOD2, and SOD3, respectively [27]. SOD1 is located at locus 21q22.11, SOD2 is located at locus 6q25.3, and SOD3 is located at locus 4p15.2. SOD1 is distributed throughout the cytoplasm and nucleus while SOD2 is found in the mitochondrial matrix [46, 47]. Interestingly, SOD3 is a glycoprotein that is predominantly expressed in the extracellular matrix of tissues and the glycocalyx of cell surfaces where it is anchored to heparansulfate proteoglycan. SOD3 has been detected in human plasma, lymph, ascites, and cerebrospinal fluids. According to the Genotype-Tissue Expression (GTEx) project, SOD3 is most abundant in the Aorta, Tibial, and Coronary arteries, which corresponds to its role as an extracellular antioxidant [48]. SOD3 and its extracellular expression are noteworthy due to the oxidative stress and subsequent paracellular permeability within the BBB (Figure 1).

The general reduction pathway for metal-coordinated forms of SOD enzymes is the following:

(2) $$\begin{array}{c} {Cu}^{2+}\text{-}SOD+{O_{2}}^{-}\to {Cu}^{+}\text{-}SOD+O_{2} \end{array}$$

(reduction of copper; oxidation of superoxide)

(3) $$\begin{array}{c} {Cu}^{+}\text{-}SOD+{O_{2}}^{-}+2H^{+}\to {Cu}^{2+}\text{-}SOD+H_{2}O_{2} \end{array}$$

(oxidation of copper; reduction of superoxide)

where the superoxide anion is converted to two less harmful products, hydrogen peroxide (H_2_O_2_) and dioxide (O_2_).

While SOD2 and SOD3 have had little published linkage to disease, SOD1 has been associated with Familial Amyotrophic Lateral Sclerosis (fALS) [49], and SOD1 is implicated in apoptosis [50]. GTEX analysis shows high SOD1 representation in the brain (Figure 1). For this reason, SOD1 has been the primary focus for fALS research, but considering SOD3's expression and abundance in the blood stream, it may be a potential target for future BBB permeability research.

#### 2.1.3. CAT Protein

Encoded by the CAT gene, catalase is a tetrameric protein. Each of the four subunits is 60kDa in weight and each contains a single ferriprotoporphyrin [51, 52]. CAT is ubiquitous in living tissues that utilise oxygen. Using an iron or manganese cofactor, CAT catalyses the reduction of hydrogen peroxide to form water (H_2_O) and molecular oxygen. This process works in a complementary relationship to SOD family proteins in detoxifying the resulting hydrogen peroxide by converting it to water and dioxide (O_2_) [53].

(4) $$\begin{array}{c} 2\;H_{2}O_{2}\to 2\;H_{2}O+O_{2} \end{array}$$

The biochemical mechanism of reaction of catalase is largely speculative; however it has been proposed that it occurs in two stages [54]:

(5) H2O2+FeIII-EH2O+O=FeIV-EH2O2+OFeIV-E→H2O+FeIII-E

(Fe-E represents the Ferriprotoporphyrin centre)

**Figure 2:**
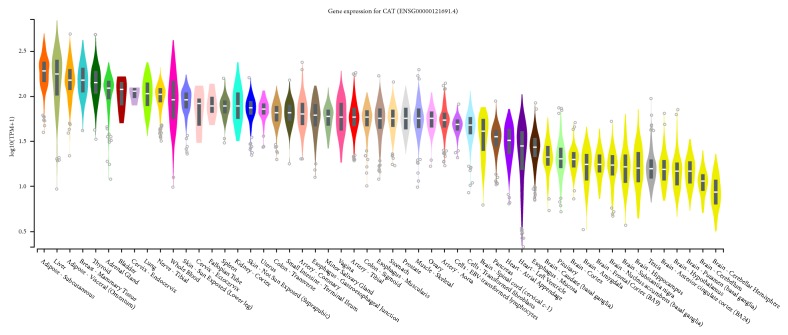
GTEx Analysis Release V7 Gene expression showing gene expressions of CAT in TPM (Transcripts Per Million) represented logarithmically.

CAT is located at locus 11p13. A mutation in this gene typically results in Acatalasemia causing oral gangrene and possibly an increase in type 2 diabetes; however studying this condition is challenging as many cases are asymptomatic and are only diagnosed as a result of affected family members. Catalase experiences the least amount of expression in the brain with slightly higher expression in the spinal cord (cervical c-1; Figure 2). An immunocytochemical study [55] showed higher expression of catalase (as well as SOD3) in the grey matter of human MND cervical spinal cords when compared to normal human cervical spinal cords. It is not known whether the high free radical scavenging enzymes occur as a compensatory reaction to the presence of oxidative stress. Further research regarding these expression level discrepancies is required.

#### 2.1.4. GPx Protein Family

Glutathione peroxidase (GPx) is the name given to a group of 8 isozymes that are present in humans. These proteins (GPx1-GPx8) have been mapped to chromosomes 3, 14, 5, 19, 6, 6, 1, and 5, respectively [33, 56–58] (Table 1).

GPx catalyses the breakdown of hydrogen peroxides into water and a lipid peroxide to corresponding alcohols [59]. Glutathione (GSH) acts as the electron donor to hydrogen peroxide and is converted to glutathione disulphide (GS-SG).

(6) $$\begin{array}{c} 2GSH+H_{2}O_{2}\to GS\text{–}SG+2H_{2}O \end{array}$$

Due to the widespread nature of GPx enzymes, their clinical importance has been explored with lower GPx activity being associated impaired antioxidant protection [59]. Despite its many forms, GPx is poorly understood; however it is speculated that the GPx family (especially GPx1) is essential to vascular oxidative stress and endothelial disfunction [59], both of which are critical components of a healthy functioning BBB [2].

### 2.2. Nonenzymatic Antioxidants

#### 2.2.1. Glutathione

Glutathione is a tripeptide containing cysteine, glycine, and glutamate. It is present, typically, in concentrations up to 12 mM within mammalian cells [60]; however it is among the most common soluble antioxidants in the brain [61], being produced by both neurons and glial cells with particularly high abundance in astrocytes [62–64]. Glutathione is synthesized in vivo by the action of two enzymes, *γ*GluCys synthetase and glutathione synthetase [65]. Glutathione (GSH), being a vital cellular component, acts as a redox buffer and as a cofactor for signal transduction, antioxidant and electrophile defence mechanisms, especially in the CNS. Thus, dysregulation of GSH homeostasis and deactivation of GSH-dependent enzymes are believed to contribute to initiation and progression of neurodegenerative diseases. It is thus no surprise that the alteration and dysregulation of glutathione homeostasis in glutathione-dependent enzyme activities are implicated in the induction and progression of neurodegenerative diseases, including Alzheimer's disease, Parkinson's disease, Huntington's disease, and amyotrophic lateral sclerosis [66].

Decreased glutathione has been demonstrated to promote apoptotic pathways partially contributing to motor neuron degeneration in* in vivo *and* in vitro *ALS models including elevated cellular ROS production, increases in oxidative stress marker gene expression, decreased cell adhesion, and positive association with motor neuron death and ALS-like disease progression [67].

During the detoxification of ROS, glutathione involves itself in one of two ways: reacting nonenzymatically with radicals such as superoxide anions, nitric oxide, or hydroxyl or as the acting electron donor for the reduction of peroxides in the GPx family. After being catalysed by GPx, glutathione is regenerated from glutathione disulphide by the enzyme glutathione reductase (GR) [65]. A disruption in any of the genes responsible for glutathione synthesis or regeneration would increase oxidative stress by not only the removal of glutathione but also impairing GPx action.

#### 2.2.2. Carotenoids

Carotenoids are a group of approximately 700 species of lipo-soluble C-40-based isoprenoid pigments that are characterised by their extended conjugated *π*-electron system [68]. These pigments are only synthesized by plants and microbes and as such need to be supplemented into the human diet [69]. Of the 700 identified species, only 50 have been linked to play a role in the human diet [70]. As a result of their extended conjugated structure, carotenoids have a high affinity and strong role as an antioxidant to singlet oxygen (^1^O_2_) and peroxyl radicals [71]. The reactivity with ROS is largely dependent on the number of conjugated double bonds within the carotenoid's structure: the longer the extension of the *π*-electron system, the stronger the carotenoid's antioxidant potential [72].

Carotenoids rely on either passive process or active transporters (scavenger/transport proteins) SRBI, CD36, and NPC1L1 for cellular uptake [73]. Due to the dietary source for carotenoids in humans, it is of interest to note the expression levels of these active transport systems: misfolded proteins or mutated genes for these systems may be of interest in explaining an abundance of ROS. Sequence variations of NPC1L1 proteins exist; however these have been investigated in relation to dietary cholesterol absorption [74]. While not immediately relating to MND, misfolded or otherwise nonfunctional variants of CD36 and NPC1L1 remain an area of MND and carotenoid research importance.

### 2.3. Vitamins

Vitamins are essential organic micronutrients that an organism cannot synthesise themselves. Vitamins A, C, and E are all supplemented in human diets and act as essential antioxidants [75–77]. Compromised nutrition is a well-documented issue within ALS patient populations, although most frequently this is due to difficulty eating due to dysphagia, a common symptom of ALS [78–80]. It is not known if this poor nutrition affects the severity of ALS due to the absence of documented evidence: for this reason vitamins and nutritional support are best thought of as an early adjunctive therapy rather than a late palliative therapy [81].

#### 2.3.1. Vitamin A

Often discussed alongside the structurally similar carotenoids is Vitamin A. Vitamin A is found in the body as retinol, retinal, and retinoic acid. Vitamin A can act as an antioxidant via the hydrophobic chain of polyene units that can decrease energy levels in singlet oxygen, neutralizing thiyl radicals and combining with and stabilizing peroxyl radicals [75]. Vitamin A is stored in extracellular fluids and within cells bound to proteins such as retinyl palmitate, oleate, and myristate within the liver, kidneys, intestines, and lungs [75]. Disruption of the genes responsible for Vitamin A's storage proteins may impede its function as an antioxidant.

#### 2.3.2. Vitamin C

The concentration of vitamin C in the brain exceeds that of blood by 10-fold, and in tissues, vitamin C exists primarily in the reduced form, ascorbic acid. Agus* et al*. [82] showed that the oxidized form of vitamin C, dehydroascorbic acid (oxidized ascorbic acid), readily enters the brain and is retained in the brain tissue in the form of ascorbic acid. Mechanistically, the transport of dehydroascorbic acid into the brain was inhibited by d-glucose, but not by l-glucose. To support this, the facilitative glucose transporter GLUT1, which is expressed on endothelial cells at the blood-brain barrier, is responsible for glucose entry into the brain, thereby providing evidence showing that GLUT1 also transports dehydroascorbic acid into the brain [82]. Ascorbic acid is transported by the SVCT family of sodium-coupled transporters, with two isoforms molecularly cloned, the transporters SVCT1 and SVCT2, that show different functional properties and differential cell and tissue expression. With this, Vitamin C moves throughout the body either passively or through active transporters SVCT1 and SVCT2 proteins that are encoded by the genes Slc23a1 (5q31.2) and Slc23a2 (20p13), respectively, as well as secondary active transport of ascorbate through the sodium-dependent vitamin C transporters [76]. Disruption of the genes Slc23a1 and/or Slc23a2 genes, or low sodium levels, would result in Vitamin C acting less efficiently as an antioxidant. As Vitamin C is a wide spectrum antioxidant essential for humans, which humans are unable to synthesize, it must be obtained from dietary sources, mainly fruits and vegetables [83].

#### 2.3.3. Vitamin E

Vitamin E is a group of fat-soluble molecules made up of four tocopherols and four tocotrienols. The specific function of Vitamin E is not well characterized; however major theories hold that Vitamin E (specifically the isomer known as *α*-tocopherol) is a fat-soluble antioxidant that acts primarily intercellularly in the mitochondria and endoplasmic reticulum [77]. Vitamin E acts as an antioxidant through donation of hydrogen (H) atoms to free radicals [77]. Little is known regarding the biochemical mechanisms. Foods rich in Vitamin E include many fruits and vegetables and nuts/seeds.

Across species, vitamin E has been shown to be important for normal neuromuscular function owing to its potent antioxidant nature, as well as its ability to modulate the expression of certain genes that inhibit platelet aggregation thereby stabilizing plasma membranes.

Animal studies have shown the importance of Vitamin E in a dose-dependent manner as a protective factor for BBB permeability and have demonstrated that the dose dependence of this antioxidant in aged animals differs from that in younger organisms [84]. In this context, it is worth mentioning that previous studies have shown that the deficiency in vitamin E increases brain tissue oxidative stress and impairs the integrity of the blood-brain barrier, which may have relevance to the pathogenesis of amyotrophic lateral sclerosis and other neurological diseases [85].

The underlying molecular and cellular mechanisms causing neuromuscular dysfunction as a consequence of alpha-tocopherol deficiency remain poorly understood, even though Vitamin E deficiency has also been linked with the pathogenesis of motor neuron diseases where oxidative injury plays a vital role. Here it is important to mention that the free radical damage to motor neurons has been shown [86] in the pathogenesis of ALS where several distinct mutations in the copper/zinc superoxide dismutase gene (SOD1), a critical component of cellular antioxidant defence mechanisms, have been shown [50]. Moreover, Vitamin E supplementation has been shown to slow down the progression in a transgenic mouse model of ALS [87] and more recently in a pooled analysis from 5 prospective cohort studies where long-term vitamin E supplementation was associated with decreased risk of ALS [88].

## 3. Microbleeds

Little is known regarding the prevalence and prognosis of people with cerebrospinal microbleeds. This is due to the diagnosis requiring brain MRI (Magnetic Resonance Imaging) [89, 90] and the associated costs act as a research challenge. Microbleeds can be present in individuals with no clinical history of strokes or hypertension: there is little to no understanding on observable symptoms. Similarly to physical trauma possibly affecting the BBB, the BSCB experiences disruption after spinal cord injury. Secondary injury resulting from the physical spinal cord injury can lead to apoptosis of neurons and glia [91]. Humans with ALS develop BSCB breakdown which has been reported to cause microvascular spinal-cord lesions [7]. As it stands the pathogenesis of BSCB breakdown in ALS (and wider MNDs) remains unclear and is an area for further study.

Further, there is paucity of literature describing whether cerebral microbleeding leads to BBB hyperpermeability or if BBB hyperpermeability as a result of ALS leads to cerebral microbleeds. Microbleeds are currently not known to be causative or reactionary to the conditions of ALS. Studies have linked cortical microbleeds (CMBs) with Alzheimer's disease [92, 93] and shown that CMBs, while rare in frontotemporal lobar degeneration, are increased in the regions affected by neurodegenerative lesions. This suggests that the progression of Alzheimer's disease promotes CMBs. Understanding the relationship between cerebrospinal microbleeds and MND represents a significant area in understanding the conditions and may provide a possible mechanism of prevention.

## 4. Immune Invasion and Hyperpermeability

Immune invasion of neural tissues is a common feature of ALS and other neurodegenerative diseases [94, 95]. In mouse models of ALS, expression levels of occludins and ZO-1 were characteristic of the disease process and were accompanied by increased ROS levels and probable remodelling of the extracellular matrix due to MMP-9 upregulation [96]. Uptake of Evans Blue dye by spinal regions in SOD1 mice and rats has also been shown [97, 98] and BBB hyperpermeability has also been shown in human ALS patients [16].

It is known that disruption to the BBB facilitates easier leukocyte invasion into neural tissues [99]. The types of immune cells that infiltrate areas of degenerating neurons are also highly relevant. Autopsy of human ALS patients, for instance, showed infiltration of degranulating mast cells and neutrophils along the motor pathway and within degenerating muscle fibres specifically at the neuromuscular junction [100]. Inhibition of these cells using tyrosine kinase inhibitors prevented infiltration of neural tissues in SOD1 rats and was able to preserve these structures to some extent [100]. T cells are also able to infiltrate spinal tissues of ALS mouse models and human patients [101–103] and it also appears that the differentiation fate of T cells is biased in ALS patients, as regulatory T cell numbers are decreased in ALS patients, and this decrease correlated with disease severity and rate of progression [104]. However, it is still largely unknown what roles various infiltrating T cell populations have in human ALS patients. Further experiments that aimed at determining the prevalence of T cell subsets in invaded neurological tissue would greatly aid in understanding the role of these cells in BBB disruption.

Current thinking suggests that disruption of the BBB is an early event in ALS pathogenesis, but it remains unclear if this is a disease driving factor or if it is a symptom of some other ALS core pathology. Another question which remains is whether or not other protein aggregation types can lead to this disease phenotype. Given that TDP-43 aggregates are present in approximately 97% of all ALS cases [105], this is worthy of future investigation.

## 5. Epidemiology and Underlying Insults in the Impairment of the BBB

### 5.1. Global Epidemiology

Across the world, the statistics for MND are uniform in the distribution amongst age and sex. In general, males are more susceptible than females, with peaks in prevalence in MND cases in people within the age range of roughly 50-60 years old [106]. These age-related peaks in MND prevalence correspond with the morphology of aging brains and age-associated neurodegenerative diseases such as Alzheimer's disease and dementia [107] through proposed mechanisms such as amyloid-*β* clearance [93]. Studies have shown that “agricultural or manual workers” in Eastern India had a higher correlation with ALS than other MNDs that did not discriminate [108]. Similarly, in a geographical epidemiological study of MND patients in Lancashire and South Cumbria, England, there were incidences of clusters of MND patients within towns with chemical manufacturing and outlying rural areas [109]. These studies reflect a general trend of MND clusters occurring in communities as a result of different occupational and lifestyle exposures, leading to progressive damage resulting from toxic exposure and potentially common genetic elements.

In 2016 [106, 110], the averaged worldwide all-age prevalence of all MNDs was 4.5 incidences per 100 000 people. Socio-Demographic Index (SDI) is a composite measure of income per capita. It was found that, in 2016, high SDI made up 48.9% of MND cases, high-middle SDI countries made up 15.2%, middle SDI countries made up 21·4% of cases, low-middle SDI countries made up 11·8% of cases, and low SDI countries made up 2.6% of cases. These statistics may be skewed due to different effectiveness of diagnostic information available in lower SDI countries; however assuming the data is accurate, analysing lifestyles (diet, industrial practice, exercise, and regular activities) of individuals residing within higher SDI countries would be of interest to research. The highest all-age prevalence of MND was observed in North America, Australasia, and Western Europe, while the lowest all-age prevalence was seen in central sub-Saharan Africa, eastern sub-Saharan Africa, and western sub-Saharan Africa. From this study, half of the world's prevalent cases were in countries within the highest SDI quintile.

### 5.2. Guam Studies

The high rates of ALS and ALS-like conditions in Guam are of research interest. The incidence of this condition within Guam was at one stage over one hundred times the incidence rate of ALS on the mainland United States with similarly high incidence rates of a form of Parkinsonism virtually confined to the native Chamorro population [111]. With no clear genetic pattern, Chamorro lifestyle and diet were considered, specifically ingestion of food products derived from the false sago palm,* Cycas micronesica.* The toxins from this plant have been thought to be *β*-methylamino L-alanine (BMAA), a toxic amino acid from the cycad nut, and glycoside cycasin, a known carcinogen within the cycad nut. This mechanism acts as a “slow toxin” with glycoside cycasin damaging DNA in vulnerable neurones with BMAA acting as a degenerative agent [112]. Despite being relatively ineffective at crossing the BBB, free BMAA has the ability to cross the BBB [113] although other proposed mechanisms include neutral amino acid carriers [114, 115], cerebral capillary transfer [116], and protein incorporation of BMAA [117]. With both neurodegenerative capabilities and ability to cross the BBB, BMAA represents an interesting area of study.

Despite the Chamorro population routinely treating the cycad nuts as their association with ALS is clear cases persisted leading to the idea that it was not direct consumption of these cycad toxins causing ALS; however consumption of flying foxes (*Pteropus tokudae* and* Pteropus mariannus*) have bioaccumulated these cycad toxins. Despite substantial observational evidence, this proposed mechanism has been routinely challenged [118] and the exact nature of the cause of ALS in Guam is still widely debated. Investigating the mechanism of BMAA perturbance is of interest to MND research as the mechanism could provide insight to other potential mechanisms of BBB and BSCB disturbance.

### 5.3. Circadian Clock

The Circadian Clock is an endogenous, oscillation of biological processes dependant on a molecular clock [119–121]. It is thought that the mechanisms associated with the Circadian Clock also affect permeability of the BBB. This Circadian rhythm has been modelled in* Drosophila melanogaster [121]* where levels of leptin and cytokines differed at various times of the day [10, 122]. Humans, like* D. melanogaster*, are diurnal (awake during the day, asleep at night) and therefore would presumably display similar levels of increased BBB permeability (BBB hyperpermeability) at night. The advantages of this pattern are two-fold. During active hours in which the organism is moving around encountering many different xenobiotics, the BBB is less permeable to these insults. During inactive hours, however, the organism has an opportunity to expel accumulated endogenous particles (such as amyloid beta [123]) which have built up during the wakeful hours. This suggests that increased human activity during times where the BBB increases permeability (e.g., night-shifts) or increase in the intake and production of ROS (e.g., smoking at night increasing blood carbon monoxide levels) could potentially be disrupting this Circadian rhythm, damaging the BBB through oxidative stress or other means. Evidence from a mouse model suggests that this is the case with REM sleep deprivation which leads to BBB hyperpermeability [124].

This research into circadian rhythms in humans has resulted in the definition of an individual's Chronotype: the Circadian preference of an individual, describing their proclivity for earlier or later sleep. Chronotypes vary from person to person. The well documented chronotype regulatory genes include RGS16, PER2, PER3, PIGK/AK5, INADL, FBXL3, HCRTR2, and HTR [125–127]; however as of a 2019 GWAS analysis of 697 828 individuals, the number of genetic loci associated with chronotype has increased from 24 to 351 [128]. The information contained within these genes may help identify chronotypes that confer increased susceptibility to ALS and allow us to explore the possibility that standardized working hours over vastly differing chronotypes within a population may help explain increasing rates of ALS. It is worth examining experimentally the chronotype regulatory genes in ALS patients and their impact on BBB integrity, expanding upon current ALS genes of interest. Understanding individual chronotypes and how they affect BBB permeability may allow us to identify new therapeutic mechanisms and provide a new angle of research.

It is of interest to note the multiple pathways that correspond to central nervous system and brain development, components of neuronal cells (synapses, axons, and dendrites), as well as neurogenesis within the identified chronotype regulatory genes. Again, it is of interest to review the correlations between chronotype and psychiatric traits. The most substantial of these was a positive correlation between subjective “wellbeing” (as a self-reported phenotype within the study) and being a “morning person.” There existed negative correlations between being a morning person and schizophrenia, depressive symptoms, major depressive disorder, and intelligence [128].

### 5.4. Schizophrenia

Schizophrenia is a mental illness that disrupts the functioning of the human mind with intense episodes of psychosis and periods of apathy, followed by otherwise normal mental functioning. While often spoken of separately, there is biological and epidemiological evidence for an association between those suffering motor neuron disease and psychotic illness, particularly Amyotrophic Lateral Sclerosis (ALS) and Schizophrenia. A genetic correlation of 14.3% [129] has been placed between ALS and Schizophrenia. Furthermore, the presence of the (apparent) monogenic C9orf72-driven overlap and polygenic overlap in the aetiology in ALS and Schizophrenia patients suggests the presence of a common biological process [129]. Interestingly, there have been propositions that prolonged use of antipsychotic medication may prevent ALS sufferers from developing all/some clinical features of the illness [130]. These antipsychotic/antidepressant medications include Chlorpromazine [131] and Clozapine [132, 133]. While ALS cannot, at this stage, be cured with antipsychotics/antidepressants, there appears to be some therapeutic relevance for their use in ALS, but their use in ALS patients still remains controversial at best.

The research suggesting linkage between ALS and Schizophrenia is in its infancy and does not suggest a causative relationship between the two conditions [129]. It is however of epidemiological interest as the linkage between the conditions [134, 135] may provide insight regarding the inheritance of ALS as well as widening the knowledge in terms of treatment for both disorders. This is particularly urgent given that these links have not been explored since the mid 1970s.

### 5.5. Telomere Length

Telomeres are regions of repetitive nucleotide sequences at the ends of chromosomes which serve as a protection against chromosome shortening in chromosomal replication in cell division: telomeres act as a disposable buffer such that the genetic material that is lost is nonessential [136]. Telomere length is thought to be a general function of age; at birth telomeres are roughly 11 kB [137] long while, in old age, they can be less than 4kB [138]. Generally, men have a higher average rate of decline than women [139].

To compensate for shortening telomeres the ribonucleoprotein, telomerase, extends the telomere ends of a chromosome on the 3′ end. Telomerase is comprised of telomerase reverse transcriptase (TERT), telomerase RNA component (TERC), and dyskerin (DKC1) [140]. There is a growing body of evidence suggesting that TERT deficiency potentiates BBB dysfunction and that increased TERT expression may be a result of oxidative stress resistance [141]. This is of importance as there is in vitro evidence suggesting that oxidative stress-mediated DNA damage is a significant aspect of telomere shortening.

Telomere shortening and telomerase are of interest to the not only links with oxidative stress but also unrepaired telomeres and their persistent DNA damage response signalling which is essential to the establishment of senescence [142]. An accumulation of senescent vascular cells is associated with a compromised BBB [143].

## 6. Current Concepts and Developments in Gut Microbiota and BBB Connection

The gut-brain nexus is vitally important for our general wellbeing. There appears to be an intrinsic connection between the GI tract and the brain, and any impairment of these connections between microbiome and the gut is associated with the etiology of many diseases that afflict mankind and almost all the neurodegenerative disorders [144]. Notably, the longest nerve in the human body, the vagus nerve, which extends from the brainstem to the lowest viscera of human intestines, maintains the communication and connectivity between the gut and brain. There are three recognized mechanisms involving immune activation, modification of the autonomic and sensorimotor connections, and the regulation of the neuroendocrine pathway through which the microbiome influences the gut–brain axis. Hoyles et al. [144] hypothesized that the interactions between circulating gut-derived microbial metabolites and the blood–brain barrier (BBB) could contribute to the gut–brain axis and showed that the propionate produced from dietary substrates by colonic bacteria is able to stimulate intestinal gluconeogenesis and is associated with reduced stress behaviours, but its potential endocrine role remains to be addressed.

The gut microbiota has been acknowledged for its importance in gastrointestinal development, barrier integrity and function, metabolism, immune responses, and CNS development. The development of the CNS includes formation of the BBB: an essential component in neonates during critical periods of foetal brain development [145]. It has been shown in mouse models that the maternal gut microbiota can influence prenatal development of the BBB by causing hyperpermeability in foetal mice BBBs with germ-free mothers [129]. It has also been shown that the lack of normal gut microbiota in germ-free mice is associated with a hyperpermeability of the BBB [146]. Supporting these observations, studies in humans have also revealed altered microbial profiles in children with Autism spectrum disorders (ASD) [147, 148], and oral treatment of autistic children with the vancomycin led to effective suppression of some of the gut microbiota involved in disease manifestation showing dramatic improvement. Interestingly, regression to autistic symptoms was seen upon the cessation of treatment with vancomycin [148].

In the context of hyperpermeability of the BBB, it has been hypothesized that this observed increase in permeability of the BBB may be the consequence of disorganised tight junctions and low expression of the transmembrane tight junction proteins claudin 5, occludin, Zo-1, Zo-2, efflux transport (P-glycoprotein and ABCG2), nutrient transport (Glut-1, Mct-1, and Lat-1), and others (basigin and carbonic anhydrase 4) [149]. Despite observational evidence, the precise signalling mechanisms between gut microbiota and maternal gut microbiota which modulates the BBB function remain poorly understood. It is of interest to note the current research efforts made in highlighting potential linkages between schizophrenia/other psychiatric disorders and the gut, oropharyngeal, and mucosal microbiomes. While this research is still in its infancy, previous linkages between brain development and psychiatric disorders/BBB hyperpermeability may provide some insight to either of or both conditions.

### 6.1. Mechanistic Insights on BBB Integrity/Permeability from Transcriptomics: Intrinsic Role of Microbial Metabolites

Recent genomic and proteomic studies of the BBB have identified unique molecular traits of this vascular bed, and the emerging concepts suggest that the BBB is heavily involved in brain function [150]. A number of animal and human studies support the existence of an intrinsic gut, brain communication, although the underlying mechanisms remain poorly defined. Accumulating evidence suggests that the gut microbiota is able to influence the integrity and permeability of the BBB, which is supported by hyperpermeability and dysregulation of interendothelial cell tight junctions in both antibiotic-treated and germ-free mice exhibiting considerably enhanced barrier permeability [151]. Interestingly, upon conventionalization, these impairments could be reversed. The mechanism(s) by which gut microbes are able to exert their influence remains unclear, but alterations in gut microbiota changes that can lead to consequential changes in brain chemistry independently of vagal or sympathetic neural pathways and in the absence of any immune response do suggest a role for soluble gut metabolites in BBB hyperpermeability [152].

The aforementioned data does highlight a likely role of short-chain fatty acids (SCFAs), which may be acting as key microbial mediators influencing the gut–brain axis, although the majority of studies looking at SCFAs for their role in the gut–brain axis largely focused on butyrate [153], as opposed to relatively fewer ones that investigated the role of propionate, a highly potent FFAR3 agonist.

It is to be noted that both butyrate and propionates show comparable plasma concentrations and receptor affinity, and increased intestinal propionate was found to be associated with reduced stress behaviors in both mice and humans. Thus, its role in endocrine mediation in the gut-brain axis remains underexplored. Moreover, given the presence of FFAR3 on endothelial cells, Hoyles et al. [144] hypothesized that propionate targeting of the endothelium of the BBB would represent an additional facet of the gut–brain axis and explored using transcriptomics to study the effects of exposure to physiological levels of propionates to BBB. Treatment with propionates showed significant differential expression of 1136 genes of which 553 were upregulated and 583 were downregulated [154].

Mainly the protein processing in the endoplasmic reticulum and RNA transport pathways were activated upon exposure of cells to propionate, in addition to gene expression in pathways associated with nonspecific microbial infections that were inhibited by propionate. Other affected pathways were the cytosolic DNA-sensing pathway usually upregulated by pathogen DNA during microbial infections, thereby triggering innate immune response, NF*κ*B, and the Toll-like receptor signaling pathways. Of the 19,309 genes examined, 203 of the 224 genes were found to be associated with the BBB, and 11 of these DE genes were significantly associated with the inflammatory response, implying inflammation to be one of the key events in BBB hyperpermeability, which has a crossover between all the known NDs. Using hCMEC/D3 cells treated with TNF*α* and IFN*γ*, Reijerkerk et al. (2013) profiled the micro-RNAs that modulate BBB function under inflammatory conditions. With a panel of miRNAs identified to have differential expression induced by inflammatory mediators that also displayed opposing changes after barrier induction in the cell line, the authors further examined their expression in brain capillaries isolated via filtration from resected multiple sclerosis patient lesions. These samples showed decreased expression of miR-125a-5p [155]. miR-125a-5p is a key regulator of brain endothelial tightness and immune cell efflux, and these findings suggest that repair of a disturbed BBB through microRNAs may represent a novel avenue for effective treatment of MS and can be applied to other neurodegenerative diseases. Thus, finding miRNAs that regulate the expression of genes that are unique to the BBB microvasculature may open doors to unveiling how inflammation is governed in this immune privileged site [155].

It is important to iterate that, since the SCFAs are not able to fully recapitulate the BBB-restoring effects of conventionalization of germ-free animals, as shown by Hoyles et al. [144], it has been hypothesized that additional circulating gut-derived microbial mediators are possibly contributing to the regulation of BBB function, which warrants further investigation. To date >200 different microbial metabolites are known to exist in human blood circulation [156]; thus there is clearly a great potential in studying these metabolites for their role in gut dysbiosis, gut and brain communication, and changes in these metabolites in healthy vs diseased individuals, in addition to their influence on the levels of these metabolites on BBB integrity and hyperpermeability. These data will prove to be highly meaningful in understanding the development of neurological diseases, as variability in BBB function is increasingly being recognized across all NDs and cognitive impairment. Thus, the knowledge of tissue-specific gene expression at the BBB and identification of genes, their expression, miRNA, and proteome could provide new targets for drug delivery across the BBB and lead to the elucidation of mechanisms involved in brain pathology at the microvascular level. In this case more human studies seem warranted.

## 7. Conclusions

This review aimed to provide insight into the physiological, biochemical, and novel genomic mechanisms behind BBB hyperpermeability. Not only is this topic poorly characterised within literature, but also there is little overarching direction behind research efforts. Many papers were omitted from this review due to there being a lack of wider literature support or reliance on speculative evidence. A wider goal of this review was not only to provide insight on but to present potential research targets to fully characterise BBB hyperpermeability.

Oxidative stress appears to be a prevailing idea within literature of BBB hyperpermeability despite the mechanisms not being completely realized. Further researches into the mechanisms of hypoxia and reoxygenation are needed for this to be a more definitive lead. Furthermore, research into not only genes responsible for encoding antioxidants but the mechanisms surrounding triggering events such as “oxidative bursts” which stimulate production of antioxidants would be of importance in identifying areas for further research. It would be worthwhile to conduct further research into storage and active transport systems of nonenzymatic antioxidants as they may also be of interest. Unfortunately, antioxidant therapies are not capable of curing or halting ALS in humans or animal models currently, but they have been shown to increase motor performance across SOD1 mutant mouse studies [157]. It is currently suggested that antioxidants be used as adjunct therapies [157], and with a deeper understanding of oxidative processes that drive BBB hyperpermeability in ALS, perhaps antioxidant therapies can be made more effective.

Further investigation into cerebrospinal microbleeds is worth the effort given associations of microbleeds with BBB hyperpermeability in ALS and other neurodegenerative diseases. Epidemiology of MND and the BBB is surprisingly of little substance. There is much literature on very specific aspects of MND incidences; however very little correlation between works has been done. Most epidemiological studies have relied on hospital data in country: country comparisons of MND prevalence. This is a concerning feature as many symptoms of MND can be mistaken for others and different countries have different funding allocated to hospitals resulting in better/more reported cases because of superior diagnosis.

Research into Circadian rhythms in humans and their relation to the BBB is novel and potentially links lifestyle factors to oxidative stress for specific reasons; e.g., all smokers may not have an increase in the permeability of their BBB; however, night-time smokers [158] may increase oxidative stress when the brain is vulnerable [119–121], leading to a hyperpermeable BBB, leading to MND.

Telomere length is a promising area of study to look at considering the findings in shortened telomeres almost reflect cases of MND; i.e., men are more affected than women, and older populations are more affected than younger ones. Telomeres and their roles in oxidative stress are of great interest to pursue.

Lastly, almost all neurological disorders maintain a profound association with the BBB, and any alterations in the microvasculature of the brain lead to inflammation and hyperpermeability. Understanding the role of genes, proteins, and miRNA that form the BBB vasculature is vital to understanding the processes BBB governs during disease and will explain underlying reasons for the disruption of the BBB during infectious and neurodegenerative diseases. Small molecules, such as miRNA, can not only lead the way in identifying novel biomarkers, but may also explain how the gene and protein expression is regulated in the BBB by miRNAs.* Reijerkerk* et al. [155] have suggested that therapeutic application of microRNAs, such as miR-125a-5p, could potentially reestablish normal functioning of the brain vasculature in endothelial cell-based neurological diseases, in particular MS, and can be applied across NDs if we identify miRNAs in BBB.

## Figures and Tables

**Table 1:** Known GPx protein family and their chromosomal location.

Gene	Locus

GPx1	3p21.3

GPx2	14q23.3

GPx3	5q23

GPx4	19p13.3

GPx5	6p21.32

GPx6	6p21.1

GPx7	1p32.3

GPx8	5q11.2
